# Development of Dental Tissues

**Published:** 1863-06

**Authors:** 


THE
### DENTAL REGISTER OF THE WEST.

VOL. XVII.] JUNE, 1863. [No. 7.

### Original Essays and Communications.

### DEVELOPMENT OF DENTAL TISSUES.

(Read before N. Y. Society of Dental Surgeons, on the evening of June 11, 1862.)

BY W. H. A.

All bodies originate in an unseen organism that projects itself into material presence by the intervention of material substances in solution or so finely divided as to render them mobile under the fine attractions and repulsions of molecular magnetisms.

This law is well displayed in the formation of human teeth; in fact all teeth and other bodies.

Creation is instantaneous. Evolution is simply making apparent the works of creation, and therefore has especial reference to periods or stages of the work of unfoldment of the type produced in the creative act.

Properly considered creation is not limitable by time or space, and hence must be the work of infinity; the only part of whose works seen by us is that which finds means of symbolization in material shapes and sizes of corporeal being. Simple division can never reduce matter to a nonentity, however easily it may pass it out of the range of our powers of vision. The aggregation of ultimate atoms, equally with their separation, elude detection; we only perceive them after, not in the act.

All solution is but the play of affinities subsisting in and between the various physical and spiritual bodies capable of this action.

The highest type of mammatian tooth is an exact epitome of the three kingdoms in nature on this planet, in a word, a a complete microcosm in which the mineral, vegetable and animal kingdoms are infinitismally represented or contained. Viz: The enamel is the exact synonym of the rock or mineral, the dentine of the vegetable modes of nutrition, and the pulp the perfect symbol of animal life as pertains to the soft or more highly vitalized and complicated animal tissue.

I am aware that naturalists have denied organism and necessarily nutrition to the mineral kingdom, and so have anatomists denied vitality to the enamel. But, so soon as we are enabled to so far systematize the works of nature or creation as to perceive the very first basal aphorism, "all things differ but in degree we shall be able to take a step in the advance of the older naturalists and histologists.

The enamel increases in density and sharpness of definition of its columns from its incipiency to complete calcification; this much has long been acknowledged. And this admission contains all I claim, viz: that crystals have a sort of nutrition; to be sure a very low and primal form of this vital act, but nevertheless complete, in its degree, as is any of the higher expressions of this admirable agency denominated organizing force, so freely acknowledged in the complications of development, growth and nutrition in plant and animal forms of life.

The nutrition of enamel then is identical with that of crystalography and is so primal that it is not directly seen but is mentally perceived with even more of certainty than any thing can be *seen* only.

The nutrition of dentine takes place along the tracts of the dentinal tubules by an ultimate act very nearly allied to that of the enamel and is also called calcification. This process is constantly solidifying the interspaces between the tubules till that is complete and then it is continued in the distal ends of these, rendering them solid lime with a very little animal substance entangled in its hard mass. This action continues to progress toward the proximal ends of the tubes as long as the pulp retains vigor enough to bring the lime salts into their open ends on the periphery of the pulp. Upon the vigor of the pulp does not only complete consolidation of all the tubes depend, but also the ultimate destruction by calcific action of the pulp itself. When this last phenomenon is perfected the vitality of the tooth and its tolerance in the alveolus depends upon the circulation in the cementum for the nutrition of all its parts.

This last named structure, viz: cementum, is next in the order of hardness and vitality, and properly claims our attention in this place.

The nutrition of cementum is a degree more complicated than dentine and is ultimately effected by the same action as dentine and enamel, viz: calcification.

But the calcified portions are not so compact nor are their lime fibres nearly so large or dense as either of the other two; so they are not so far removed from ready solution and resolidification as those. Hence, reproductions of this substance are quite common, although but a few observers have yet verified this fact, and, therefore, it is not generally understood and acted upon, as it will be so soon as it becomes generally known how completely it is amenable to restoration under this law.

Cementum is exactly intermediate between bone and dentine, and like bone is capable of production and reproduction so long as its proper organ remains entire and healthy.

Periosteum secretes bone and a modified periosteum secretes the cementum in exact accordance with the modification which differentiates it from periosteum.

The pulp is amenable to all the changes common to the animal tissues proper. It is capable of inflammatory and other modifications of the nutrient forces resident in it; its isolated and confined situation being the principal modifying circumstances which render this structure more liable to atrophy and other forms of death, and more difficult to treat than the vascular and cellular tissues in general.

Pulp like every other agent in the universe, when active to completeness without interruption, annihilates itself by doing its work so well that it ejects itself from the field of its activity by filling this field with the products of its work (calcified pulp) to the exclusion of everything else. Thus, like all true workers, building its own monument by giving itself freely up to the fulfilment of its highest and completest function.

But so very few pulps or people thus fulfil the true intent of their being without interruption or irregular action that it is the exception rather than the rule to see pulps thus going straight on from inception to culmination. And hence we have seams and wrinkles in the products of pulp plainly indicating arrest of action, irregular action or partial fulfilment of function, so that its working power becomes exhausted often long before complete calcification has been effected. At times calcific action is so active at the apex of the fangs of teeth as to lessen the passage-way of nutrient vessels to the extent of inflammation or atrophy whereby all further supply of hardening material is cut off and we thus have consumptive teeth produced. Then these things being so does it not demand of us, every one, who attempts to operate upon teeth to pry into these things with all earnestness and ability that we are capable of commanding?

The questions of what enamel, dentine, cementum and pulp really are? and where located? and how produced and maintained in normal integrity? and how restored or substituted? are of too much importance to us and our patients to be allowed to pass from investigation and full and free discussion, with the bare allusions heretofore afforded them by nearly our whole body in this country!

The positions taken by those in good repute for intelligence and skill in our profession in the discussions involving the anatomy and histology of dental structures, at our large meetings, is tangible testimony to the dearth of knowledge prevalent on these interesting and necessary topics for full elucidation when they will constitute the doctrines upon which to base a beneficent and satisfactory practice.

Without scientific accuracy and certainty in our knowlege of principles we are all afloat in our practice at the mercy of the fancy of the moment, of little use to our patients and of no use to the science of the profession at all and alternately congratulating ourselves for shrewdness in guessing right where we make happy hits and at times almost ready to abandon our calling when we fail with disgust at the "*difficulties* that beset us," when we should lay the blame in the right place and charge our failures upon the blundering ignorance of assuming the responsibilities of a profession we had not taken the precaution to prepare ourselves to practice intelligently and successfully.

The time was when something like this was necessary but thanks to a few who have digged the way up out of this slough of despond a better way is now open for us all if we will only seek it.

